# Reflections from a breast surgeon with breast cancer on how to improve cancer care

**DOI:** 10.3332/ecancer.2019.983

**Published:** 2019-12-12

**Authors:** Elizabeth Louise O’Riordan

**Affiliations:** Retired Consultant Oncoplastic Breast Surgeon and Learning from Deaths Medical Reviewer, West Suffolk Hospital, Hardwick Lane, Bury St Edmunds, IP33 2QZ, UK

**Keywords:** cancer, communication, breaking bad news, patient choice, sex, survivorship

## Abstract

Healthcare professionals pride themselves on providing high-quality care for their patients. On the whole, we are very good at offering clinically effective and safe treatments and can benchmark our services against our peers. The one area where many providers fall short, however, is the patient experience. When a consultant breast surgeon was diagnosed with stage 3 breast cancer, she realised how much she did not know about breast cancer, and how much more healthcare professionals can help patients and their carers cope with a cancer diagnosis, both during treatment and beyond. It is not enough to tell patients what will happen to them. We need to help them physically, mentally and emotionally through every stage of cancer treatment. You can only really learn how to improve the patient experience by asking patients themselves. Here are some of the lessons this consultant learned when she found herself on the other side of the table, and how to use them to improve the care of your own cancer patients.

I thought I knew everything one should know about breast cancer. I was a consultant breast cancer surgeon. I even had a postgraduate degree in oncoplastic breast surgery, but I was wrong. It was only when I was diagnosed in 2015 with stage 3 breast cancer at the age of 40, and again with a locoregional recurrence in 2018, which I realised how little I actually knew, and how ill-prepared many patients are to face the hurdles in front of them.

I did not think I had breast cancer. I thought my lump was another cyst. After all, I should know what a cancer feels like, shouldn’t I? My mammogram was normal. When it came to the ultrasound, I turned to look at the screen, expecting to see the familiar black blob. Instead, I saw a cancer. I didn’t need to wait for the biopsy results – I knew. At that moment, my life changed forever. I knew I’d need a mastectomy. I knew I’d need chemotherapy. I had a rough idea of what my 10-year survival would be. No careful drip-feeding of information. No learning curve. And ever since that moment, part of me has been in denial. A stranger is going through the physical and mental toll of breast cancer treatment, not me.

## How do you tell someone they have cancer?

Most healthcare professionals will get formal training in how to break bad news, using roleplay with experienced actors and hopefully watching their senior colleagues do it. But this has to be one of the most awkward experiences you will ever face. The first time I heard someone being told they had cancer, I just wanted to shrink into the corner of the room and disappear. I felt I was intruding on an incredibly private, emotional and traumatic experience and had no right to be there. But I needed to be there, to listen to the phrases the consultant was using, look at their body language and learn how they handled the raw emotion of the patient and her partner. You think you know how you will do it when the time comes. The words you will use. How you will sit. How empathetic and kind you will be. But nothing prepares you for the first time. Dealing with the responsibility of changing someone’s life forever, and not in a good way. Learning that those well-planned words may not actually work for you, and that patients react in many different ways – from tears to physical anger to indifference, and what you do with the emotions that you’ve absorbed during the day. It’s hard.

Over half of my time as a breast surgeon was spent in the clinic, and most days, I would end up telling many women that they had breast cancer. It often felt like I would break a woman, pick up the pieces, pass her on to the breast care nurse and repeat. It became routine – I had a lot of information to give (what cancer they have, what treatment they need, whether they might need chemotherapy and radiotherapy, the complications of all those treatments), and several boxes to tick – dates for surgery, pre-op assessments, follow-up clinics to be booked. The constraints of the NHS mean that all of that has to be done in one clinic visit, right after telling the patient that they have cancer.

I thought I was good at telling people they had cancer. But until you have been on the other side of the table, you have absolutely no idea what impact your words have. Suddenly, it was my turn to sit in a waiting room, clutching my husband’s hand so tightly that it might break, staring at the floor because if I looked at him, I’d start crying, knowing I was about to be told I had cancer. I didn’t realise what a huge deal getting bad news is. I remember every single detail of every ‘bad news’ conversation I had as a patient. Every detail: What I was wearing; What was said; How my doctor reacted; How my husband reacted and What the weather was like. I could give you the exact time of day if you asked me. I used to tell women with small cancers that they were lucky we caught it early. No one is lucky to have cancer. The words you use as a doctor take on a different meaning when you’re hearing them for the first time as a patient.

Instead of being able to focus on all the information I was being given, all I kept thinking was ‘*insert your own swear word* – I’ve got cancer’. When it was over and I had been given a plan, all I wanted to do was to run out the room screaming. However, like most patients all over the world, I had to do the walk of shame past everyone else waiting to be seen, down long hospital corridors, desperately swallowing to stop myself from screaming and shouting and swearing, until we got to the car and I could finally howl at the unfairness of it all.

## Do you help your patients explain cancer to their loved ones?

For me, one of the hardest things was telling my family that I had breast cancer. I knew too much, and they knew very little. How do I just become a patient? Everyone asks you questions – ‘What’s your prognosis?’, ‘Will you need chemotherapy?’, ‘When do you get the results?’. The list goes on. I didn’t realise that patients are forced to be experts when they’re only just getting to grips with the diagnosis themselves. It was easy for me to answer their questions because I’m an expert in treating breast cancer, but I was talking like a doctor to a patient, and not as a patient myself.

The medical profession is generally very bad at signposting patients to the information they need. I picked up every leaflet I could find in the Macmillan centre – and didn’t read any of them. I found all the information I needed online and realised I could e-mail the pdf of the chemotherapy leaflet to my mum, instead of reading it out over the phone. The last thing patients need is to have to explain what is going to happen to them to everyone they speak to. All I did was Google for the answers and pass them on because I felt I couldn’t tell my close family to just Google it themselves. We should tell patients where to direct their family and friends to get medically accurate information – such as Macmillan (https://www.macmillan.org.uk) and Breast Cancer Now (https://breastcancernow.org) websites which also have helpful hints for carers and family members.

## Who would you choose to treat you?

One of the first things my surgeon asked me was where I wanted to be treated. She was my trainer, my mentor and a friend, and she wasn’t sure she could be my surgeon. It’s very hard to treat colleagues, especially those that you are close to. I’m lucky in that I know by name or reputation most of the breast surgeons in the UK. I could literally have my pick – but how do you decide? Is a good bedside manner more important than surgical skill? Is a centre of excellence like The Royal Marsden or The Christie better than a small DGH? How far are you prepared to travel to get what you think will be the best treatment? It was a relatively easy decision for me – I stayed with my surgeon. She was brilliant and she was local and I trusted her. But that came at the cost of losing our friendship so she could remain impartial and act as my surgeon and not my friend. I’m willing to bet that most people reading this wouldn’t be happy to be treated by anyone in their department. There will be someone that you wouldn’t chose, for whatever reason. Patients don’t have that inside information. What do we, as healthcare professionals, do about it? They say that the standard you walk past is the standard you accept, but it’s very hard to tackle colleagues who are underperforming, especially if it’s their personality or bedside manner that needs improving and not their knowledge or skills. I still don’t know what the answer is.

## We tell patients what will happen to them, but we don’t tell them how to cope

Because my cancer was large and I was young, I had chemotherapy first. I thought I knew what the side effects of chemotherapy were as I’d been counselling patients for years. But when you’re in the hot seat, having a long, scary list read out to you that you have to agree to and sign, it’s completely different. Agreeing to have a treatment with serious, potentially permanent side effects that could actually kill you when 2 weeks ago you were a healthy triathlete is tough. I was given leaflets explaining all the possible side effects, but I didn’t really understand how to cope or what to do when they happened. You know you’re meant to feel rotten, but you don’t know how long you wait until you should call for help. And like most doctors, I was a terrible patient, thinking I should be able to treat myself. I learned that lesson the hard way.

Chemo was a shock. The loss of pride and dignity dealing with a body that was betraying me because of the drugs being pumped into it. The drugs and injections and laxatives I had to take to combat the side effects. Sitting crying in pain on the toilet after 10 days of severe constipation. Waking up in the middle of the night thinking I’d wet myself when it was just sweat trickling down my leg from a hot sweat. Sitting in the day unit surrounded by other patients who were a good deal older than me with silent tears rolling down my face. Feeling sorry for myself because I was so young, and life wasn’t meant to be this hard. Feeling sorry for the older patients having chemotherapy – I was struggling, and I was fit and healthy going into this, so how on earth were they coping? It’s surreal that 4 years on I can’t remember what chemo was like. Every time I get a cold or get a hangover I get a brief reminder, but my mind has protected me from what I went through, and I’m grateful. I still have a daily reminder every time I look in the mirror or look at photos taken during the time, but it gets easier to accept that it was all in the past.

It was other breast cancer patients who saved my life during chemotherapy. They told me what toothbrush and toothpaste to use for my bleeding, ulcerated gums. What toiletries to use when your skin is burning and perfume makes you gag. What to eat and drink when you lose your sense of taste. How to cope with the night sweats and hot flushes. What websites, blogs and forums to use. The list goes on and on. If you’ve never had cancer treatment, you have no idea what your patients need to do to cope with that treatment. Here’s an example – I had no idea that the charity ‘Mummy’s Star’ (https://www.mummysstar.org) existed before my diagnosis. It helps women and their families who are diagnosed with cancer during pregnancy or the first year after birth and is a fantastic resource. I felt awful that I had treated women who got breast cancer when they were pregnant and couldn’t direct them to the website. It’s one of the reasons why Professor Trisha Greenhalgh and I wrote ‘The Complete Guide to Breast Cancer: How to Feel Empowered and Take Control’ [1] to share all the wisdom we’d learned and make life easier for other cancer patients.

One of the quickest ways any healthcare professional can improve cancer care is asking patients what worked for them. What are the top ten things they would say to anyone newly diagnosed? What are the websites and apps they found the most helpful? Digitally signpost your patients to safe sources of information to prevent them from scouring the Internet in the middle of the night, sick with worry. Create a list of helpful hints and tips to give patients and their families.

## It’s hard for cancer patients to make decisions about their body

I needed a mastectomy and deciding whether to have a reconstruction was so, so difficult. It’s something my patients have to decide in a matter of weeks and I didn’t understand how hard it can be. I would give them 100-page booklets full of complex information about all the different options and complications and ask them what they wanted to do. Surely it’s a relatively simple decision? It’s not. Far from it. You see, most women don’t think about what their breasts mean to them – whether they define their sexuality, their body image, could they live without them. By the time you get breast cancer, it’s too late. You can’t think rationally or logically because that breast now has cancer. And how you feel on the day of your diagnosis may be very different from how you feel months or years later when you’ve slowly come to terms with what has happened to you.

I felt ashamed that vanity was the main reason I wanted reconstruction. I couldn’t imagine throwing out half of my wardrobe because the full bra I’d have to wear to cover a prosthesis would be visible. When my cancer came back on my chest wall last year, I had to go flat. I remember walking around Debenhams crying as I looked at the only bra they sold in my size that would fit a prosthesis, mentally throwing away all the beautiful lingerie sets I had at home. It turned out that I couldn’t wear a bra because of painful scar tissue so I had no choice but to walk around lopsided.

## Take the time to learn about every treatment you prescribe to your patients

I’ve already written about how clueless I really was regarding chemotherapy. However, it was radiotherapy that was a real eye-opener for me. The vast majority of my patients need radiotherapy. I’m embarrassed to say that I had never seen a radiotherapy machine or what happened to my patients during treatment. I’d heard my previous bosses describe it as ‘just like an X-ray treatment’. They were wrong. It drained me physically and emotionally. The 3-hour round trip took its toll. Lying on the table, topless and exposed with my arms above my head, shivering in the cold room made me feel vulnerable, exposed and sad. Almost every day another silent tear would roll down my cheek once the wonderful radiotherapists left the room to start the session. I just felt so sorry for myself and for all the other people in the waiting room who would follow me, especially the treatment.

Radiotherapy was followed by Tamoxifen tablets. I used to gloss over the menopausal side effects, saying that ‘most women find they settle within 6 months or so’. Who was I kidding? The menopause was horrific – it happened overnight. Suddenly, I was dripping in the middle of the night, getting hot flushes on the hour, unable to multi-task or remember three words strung together. I realised that breast surgeons aren’t taught how to help women who are suffering and that most GPs don’t know what to do when they can no longer prescribe HRT. Again, Twitter came to my rescue and a wonderful oncologist sent me his crib sheet of drugs that can help various symptoms (all listed in our book) and things did start to settle.

I also needed Zoladex injections to switch off my ovaries. I knew that it was a monthly injection in the stomach, but I’d never seen the size of the needle. It was huge (like a brown Venflon). I spent hours looking for blogs that would tell me what it would feel like and what the side effects would be. Someone recommended asking for topical local anaesthetic cream, but I was told that it didn’t really hurt and I shouldn’t need it. They had obviously never had the injection! I’m slim so there wasn’t much fat to grab, and every time the nurse would look at me and sigh in despair.

## There’s more to life after treatment than yearly scans

There is no point in any of us treating cancer patients to the best of our ability if we don’t help them have a good quality of life afterwards. As a surgeon, all I was really concerned with was the appearance of the breast, symptoms of a possible recurrence and were they coping with the drugs. I used to think that the hardest part for my patients was coping with the medical treatment and when I saw them at a year to wave them off for another 4 years, they just went back to normal. Again, you don’t know what you don’t know. Survivorship, moving on, calls it what you will, is often harder to cope with than the treatment itself.

I never really thought about the other aspects of life, such as returning to work, exercising to reduce the risk of a recurrence, or sex. Thanks to the menopausal side effects of the drugs patients take for up to 10 years, the loss of libido, vaginal dryness and painful sex that can wreck relationships and ruin lives. Especially if you are young and single. I never talked about sex after breast cancer with my patients. I didn’t think it was my job too. I assumed my breast care nurses or their GP would cover it. What I now know is that no one really talks about sex after cancer. Women don’t talk to their partners about it, let alone their doctor. So many women have told me that they’ve asked their husbands to divorce them and go and find a woman with two breasts and a libido that works. It makes me sad, but I’ve said the same to my own husband.

There are things that can be done to help get some form of sex life back – vibrators, vaginal lubricants and dilators, oestrogen pessaries that are safe even with oestrogen-positive cancers, but sex will probably never be spontaneous again. And it’s not just breast cancer patients who need help. What about head and neck cancer patients who can’t kiss because they can no longer produce saliva and their mouth is too dry? Or stoma patients? A friend asked her doctor what she could use instead of a stoma bag when she had a one-night stand and he was horrified. Maybe because he couldn’t imagine himself having sex with a stoma, he never thought that his patients might want to.

Patients have to deal with the daily worry of recurrence. Is it cough or a ‘cough’? Did their doctor tell them what symptoms to look out for or do they turn to Google? What do you do if you’re worried? Do you see your GP or call the breast care nurse? How long do you wait before calling someone? Are your family and friends coping with yet another one of your silly worries? And when you do get a scan, the ‘scanxiety’ of waiting for the results, imagining the worst when everyone tells you it will be fine, is almost impossible to bear.

Anxiety, depression and PTSD can set in months or even years later when the reality of what you’ve been through, what you look like and what might happen in the future starts to sink in. How do you deal with the grief of infertility, altered body image, scars, pain, premature menopause or the financial difficulties and trying to go back to work? None of it is easy. And most of this collateral damage is hidden to the outside world. Everyone tells you that you look great – if only they knew.

It is these months and years after treatment when patients need the most support. Forums and social media groups help patients know that they’re not alone, but they don’t tell them what medical help is out there if they need it or what counselling they could access. The medical profession needs to do more to meet the ongoing holistic needs of every cancer patient. Even those with metastatic disease are still busy living until they die, and we tend to forget that.

It’s all too easy to think of patients as just another number in another busy clinic, and you can’t get attached to every patient you see. But every once in a while, try to see the bigger picture. Think about what your patients and their families have to cope with outside of the hospital environment, and what you can do to make their lives better. Sit in the waiting rooms, visit the chemotherapy and radiotherapy suites. Go to cancer charity websites and read all the information they have for patients. You might be shocked at what patients are being told. Read primary and metastatic cancer blogs. Get a feel for the emotional trauma that we put patients through every single day. Absorb the good and learn from the horror stories – there are plenty out there. It’s actually really easy to improve cancer care, and often it’s the little things that can make the biggest difference. You just have to ask the experts – the patients themselves.

## How does this translate into actually improving the quality of cancer care?

In my opinion, there are three simple things that anyone can do to improve cancer care tomorrow. First, signpost patients to safe, accurate and friendly information beyond the usual Macmillan leaflets. Recommend useful books, websites, forums and apps that they can browse with their loved ones and get the support they don’t yet know they need when they walk out of the hospital. Ask your current patients to give you a list of the sites they found helpful. Trust me, we’ve already done the hard work for you.

Second, make sure that every patient knows what the symptoms and signs of recurrence are, and what to do when they are worried. Give them the information when the initial treatment has ended and go over it at every follow-up. So many patients don’t realise that cancer can come back years later. Make sure that there is a clear local policy for getting worried patients back into the system. All too often we are bounced between specialist nurses and GPs without ever seeing a consultant, and all the time our mental anguish grows with the Internet for company in the middle of the night. Make sure that your open-access system is foolproof? Educate your local GPs so they know what red-flag symptoms need to be referred back to the clinic.

Third, empower patients to start living again. Have difficult conversations about work, sex, exercise, dealing with the fear of recurrence. Educate yourself and your team so you know where to direct patients if they do need help.

Start by asking all your team whether they give this information out to every patient routinely. Work out where the gaps are. Have a patient event and ask them to be honest and tell you what they needed to know on top of what you told them. When you introduce new information, you could easily evaluate it with a holistic questionnaire or survey handed out at follow-up. Ask your patients whether they received all the information. Ask them if they know what the symptoms of recurrence are and what to do if they have one. Finally, ask them to share the books, websites, forums and apps that helped them so you can pass on that information to all your other patients.

## Conflicts of interest

The author has no conflicts of interest to declare.

## Funding statement

No funding was received for writing this paper.
